# A Novel Nasal Continuous Positive Airway Pressure-Assisted Approach for Airway Management in a Patient With Dystrophic Epidermolysis Bullosa: A Case Report

**DOI:** 10.7759/cureus.105208

**Published:** 2026-03-14

**Authors:** Tsuwa Iwamoto, Shinichi Ishikawa, Keitaro Yoshioka, Rika Mutou, Yoshimasa Takeda

**Affiliations:** 1 Department of Anesthesiology, Toho University Faculty of Medicine, Tokyo, JPN

**Keywords:** airway management, continuous positive airway pressure, dental treatment, dystrophic epidermolysis bullosa, nasal intubation

## Abstract

Dystrophic epidermolysis bullosa (DEB) is a severe hereditary subtype of epidermolysis bullosa caused by mutations in the collagen gene. It is characterized by extreme fragility of the skin and mucous membranes, where even minimal shear stress can induce blister formation. Airway management in DEB patients is challenging because of oral scarring, limited mouth opening, and mucosal vulnerability. We report on the successful use of a modified nasopharyngeal continuous positive airway pressure (CPAP) technique for airway management.

A 13-year-old boy (125 cm, 21 kg) with recessive DEB was scheduled for dental extraction under general anesthesia. Chronic labial scarring limited mouth opening to 1 cm, making oral intubation impossible. Anesthesia was induced with intravenous midazolam and fentanyl, followed by sevoflurane inhalation while maintaining spontaneous breathing. A small-diameter endotracheal tube (3.0 mm ID) was inserted through the left nostril into the pharynx and connected to the anesthesia circuit. A 3.0 mm bronchoscope was inserted through the right nostril. With the mouth closed, CPAP at 5 cm H₂O and inspiratory pressure at 15 cm H₂O were applied, expanding the pharyngeal space and improving glottic visibility. After confirming a stable airway, rocuronium (12 mg) and fentanyl (50 µg) were administered. A 5.0 mm spiral silicone tube was advanced over the bronchoscope and successfully inserted on the first attempt. Oxygen saturation remained at 100% during induction and surgery, and extubation was uneventful with no airway blister formation.

Nasopharyngeal CPAP using a small-diameter endotracheal tube provided continuous oxygenation, improved visualization, and allowed for safe use of muscle relaxants, resulting in atraumatic, first-attempt intubation. This modified approach may be a valuable option for complex pediatric airway management in DEB.

## Introduction

Epidermolysis bullosa (EB) is a group of inherited disorders characterized by blistering and erosion of the skin and mucosa in response to minimal mechanical trauma caused by abnormalities in proteins associated with adhesion structures at the basement membrane zone. EB is broadly classified into four major types: simplex, junctional, dystrophic, and Kindler syndrome.

Dystrophic epidermolysis bullosa (DEB), one of these subtypes, results from abnormalities in type VII collagen and is associated with progressive scarring and mucosal involvement, placing patients at particularly high risk during perioperative airway management.

Children with DEB frequently require oral surgical interventions, as oral hygiene is often compromised by restricted mouth opening and mucosal fragility. Airway management in these patients is therefore particularly challenging.

Severe mouth-opening restriction makes direct laryngoscopy difficult, and even when insertion is possible, laryngoscope manipulation may generate shear stress on the epiglottic and laryngeal mucosa, potentially leading to blister formation and subsequent airway obstruction. For this reason, nasal fiberoptic intubation is generally preferred to avoid laryngoscope-related trauma [1]. However, in young children, arterial oxygen saturation may decline rapidly during apnea, particularly when airway management is prolonged or unsuccessful, necessitating meticulous planning and execution by experienced teams at tertiary referral centers [1].

The use of neuromuscular blocking agents in this setting remains controversial. While muscle relaxation facilitates atraumatic intubation, maintaining spontaneous respiration is often preferred in difficult airway management; however, vocal cord closure during intubation without adequate relaxation may, in turn, increase shear stress and mucosal injury.

Continuous positive airway pressure (CPAP) is a form of respiratory support that applies constant positive pressure throughout the respiratory cycle, thereby preventing upper airway collapse, maintaining alveolar oxygenation, and improving tolerance to apnea during tracheal intubation. In 2017, Strupp et al. [2] reported an adjunctive technique in which a nasal trumpet inserted into the contralateral nostril allowed passive oxygen insufflation during nasal fiberoptic intubation in a patient with EB.

Building on this concept, we describe a modified dual-nasal approach using a small-diameter nasally inserted tracheal tube placed in the pharynx to deliver CPAP (5 cm H₂O) combined with inspiratory pressure (15 cm H₂O). This strategy enabled objective confirmation of airway patency, safe administration of a neuromuscular blocking agent, and successful nasotracheal intubation without mask ventilation in a child with DEB, highlighting a highly selected but clinically informative airway management option for patients with severe mucocutaneous fragility.

## Case presentation

A 13-year-old boy (height: 125 cm; weight: 21 kg) with DEB was scheduled for dental extraction. He was born at 38 weeks of gestation via expected spontaneous vaginal delivery. At birth, extensive skin defects and blisters were observed on the fingers, heels, toes, and perianal region, leading to the diagnosis of DEB.

Preoperative evaluation revealed severe anemia (hemoglobin: 5.1 g/dL; pediatric reference range: 11.5-15.5 g/dL) and marked hypoalbuminemia (1.2 g/dL; pediatric reference range: 3.8-5.4 g/dL). His mouth opening was restricted to 1 cm (normal pediatric range ≥2-3 cm) due to chronic lip scarring.

The patient was admitted 12 days before surgery for preoperative optimization. A peripherally inserted central catheter was placed five days before surgery for preoperative blood transfusion. By the time of surgery, his hemoglobin and serum albumin levels were 8.0 g/dL and 3.0 g/dL, respectively. Brain natriuretic peptide levels and chest radiographs were monitored to avoid volume overload during transfusion therapy.

**Figure 1:**
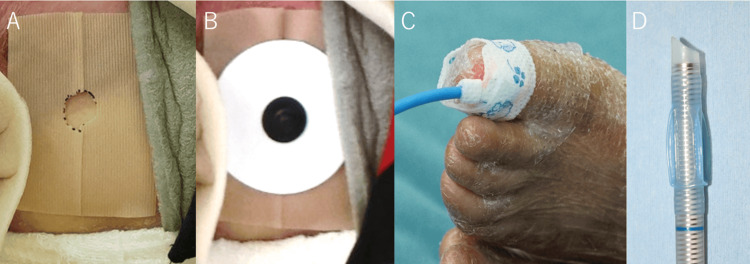
Skin protection and management for electrocardiogram (A, B), pulse oximetry (C), and silicon spiral tube (D) during general anesthesia. A. The center of Mepitac® tape (Molnlycke Health Care, Peachtree Corners, GA, USA) was rounded and filled with conductive cream used for electroencephalographic recording. B. The electrocardiographic electrodes were plastered on the above-mentioned Mepitac® tape. C. The pulse-oximetry probe was plastered on the transparent Mepitac® tape. D. Silicon spiral tube with a low-volume, low-pressure cuff which inflates at pressures below 30 cm H₂O. When deflated, the cuff forms a low, soft ridge that runs longitudinally along the tube.

On the day of surgery, no premedication was administered to avoid potential interference with airway assessment and the risk of airway obstruction associated with sedation. All monitoring devices were applied with special precautions to minimize skin trauma. Electrocardiographic electrodes were placed over Mepitac® tape (Molnlycke Health Care, Peachtree Corners, GA, USA) (Figures 1A, 1B). The central hole of the Mepitac® tape was filled with conductive cream. A pulse oximeter probe was applied over transparent Mepitac® tape (Figure 1C). A cotton dressing was placed beneath the blood pressure cuff. Eye protection was provided using flavin ointment, and Mepitac® tape was applied to the face at all points of contact with the mask and fingers. The face mask itself was lubricated with a 1:1 mixture of aqueous jelly and white petroleum jelly to minimize shear forces. After gargling with a mixture of 4% lidocaine (2 mL) and 8.4% sodium bicarbonate (0.2 mL), preoxygenation was performed until the end-tidal oxygen concentration exceeded 90%. Following two minutes of preoxygenation with 100% oxygen, general anesthesia was induced over three minutes with sevoflurane (5.0%) in conjunction with intravenous administration of midazolam (2 mg) and fentanyl (50 μg). Adequate anesthetic depth was achieved after approximately 10 minutes.

**Figure 2:**
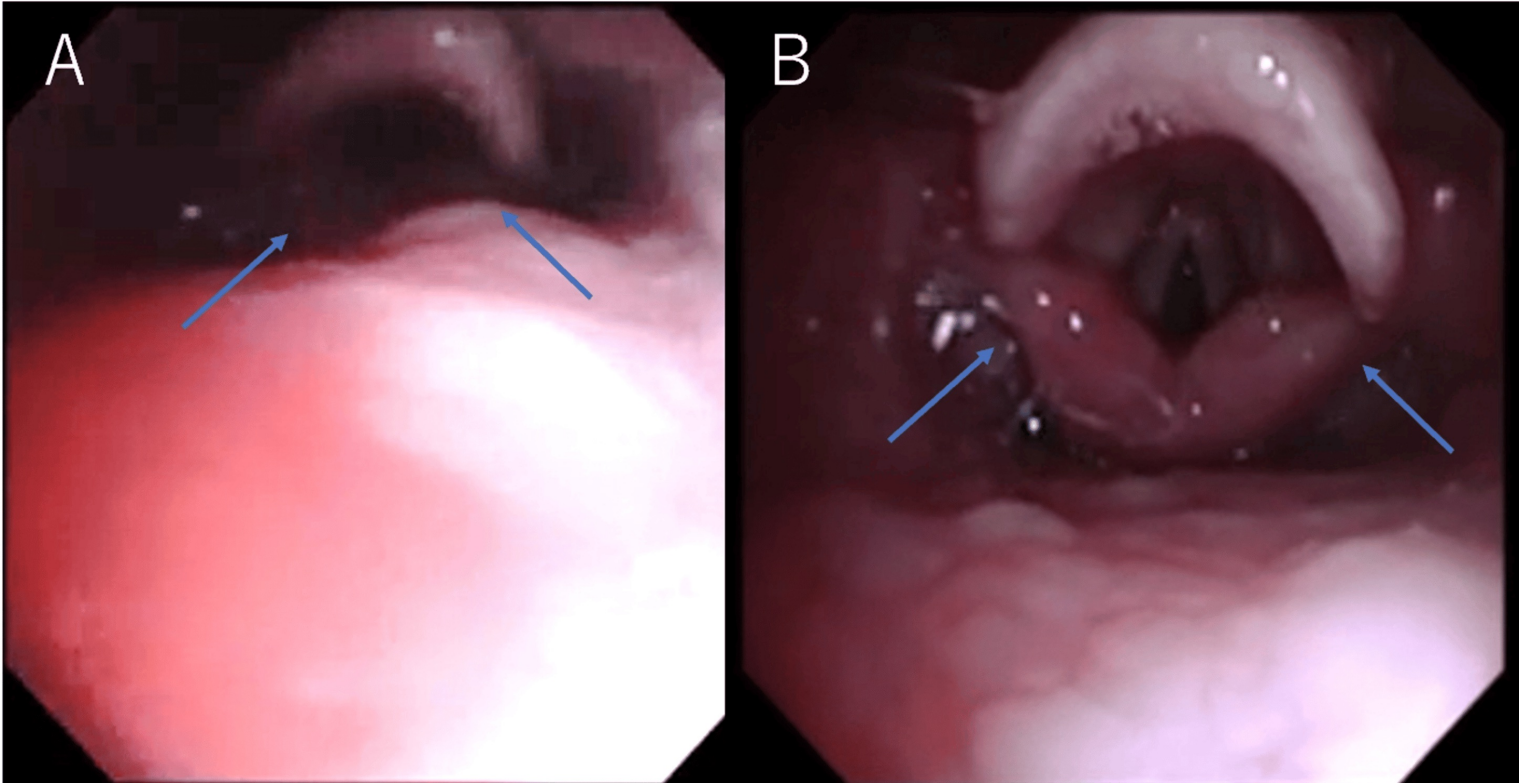
Airway management using a nasopharyngeal tube with CPAP. The application of CPAP expanded the pharyngeal cavity. (A) Without CPAP, the visualization of the pharyngeal structures from the nasal cavity was limited. (B) With CPAP (5 cm H₂O; inspiratory pressure 15 cm H₂O), the airway space expanded, allowing improved visualization of the pharyngeal and laryngeal structures. Blue arrows indicate the glottis. CPAP: continuous positive airway pressure

At this point, a small-diameter tracheal tube (3.0 mm internal diameter) was gently inserted through the left nostril into the pharynx and connected to the anesthetic circuit, where it functioned as a nasopharyngeal airway. Continuous positive airway pressure (CPAP) of 5 cm H₂O combined with an inspiratory pressure of 15 cm H₂O was applied through this tube, supporting spontaneous ventilation. Simultaneously, a 3.0-mm bronchoscope was advanced through the right nostril to allow continuous visualization of the pharyngeal structures and glottis. With the patient’s mouth manually closed, CPAP inflated the pharyngeal cavity, resulting in a stable airway and a clear endoscopic view with no active intraoral blistering, mucosal sloughing, or acute erosions observed in the larynx, epiglottis, or glottis (Figure 2).

Throughout induction and airway management, oxygen saturation remained stable at 98-100%, and blood pressure and heart rate showed no clinically significant fluctuations, indicating hemodynamic stability during CPAP and positive-pressure ventilation.

Once a stable airway was confirmed, rocuronium (12 mg) was administered intravenously. After a three-minute interval, fiberoptic nasotracheal intubation was initiated and successfully completed on the first attempt within several minutes. A 5.0 mm silicone spiral endotracheal tube with a low-volume, low-pressure cuff (Fuji Systems, Tokyo, Japan), designed to minimize shear stress, was railroaded over the bronchoscope and positioned appropriately (Figure 1D, Video 1). The endotracheal tube was secured at the nostril using a needle and thread.

**Video 1:** Smooth and atraumatic intubation assisted by a fiberscope. The bronchoscope was gently advanced into the trachea, and a 5.0 mm silicone spiral tube with a low-volume, low-pressure cuff (Fuji Systems, Tokyo, Japan), designed to minimize shear stress, was railroaded over the bronchoscope and successfully inserted on the first attempt.

The interval from the start of oxygen administration to completion of tracheal intubation was approximately 25 minutes. The dental extraction was completed uneventfully. Oxygen saturation was maintained at 100% throughout the procedure. The surgical duration was approximately 67 minutes, and the total anesthesia time was 181 minutes.

After the surgery, the patient was extubated in the operating room and transferred to the intensive care unit for overnight observation. Direct examination of the nasal cavity, pharynx, and larynx was performed using a flexible bronchoscope after extubation. No abnormal findings, including mucosal blistering, bleeding, erosion, or edema, were observed. He remained stable without airway complications, and oxygen saturation was maintained at 96-100% on room air during the 24-hour postoperative period. He was subsequently transferred to the general ward on postoperative day 1.

## Discussion

The oral cavity, pharynx, larynx, and vocal folds are lined with squamous epithelium, which is highly susceptible to blistering under shear stress. Tracheal intubation with a conventional laryngoscope can cause blistering, leading to airway obstruction after extubation. For this reason, various anesthetic techniques have been explored over time. LoVerme and Oropollo reported the use of regional anesthesia or intravenous ketamine sedation without tracheal intubation, even for complex surgical procedures [3]. James and Wark [4] reported 309 general anesthetic procedures in 33 patients with DEB. Airway management was achieved using a face mask in 48% of cases, orotracheal intubation in 37%, and nasotracheal intubation in only 6%. Intubation was described as difficult in 51% of cases, mainly due to restricted mouth opening or dental abnormalities. More recently, Brooks Peterson et al. [1] described 202 anesthetic procedures in 37 patients with EB, in which nasotracheal intubation was performed in 79% of cases under spontaneous respiration and deep anesthesia, primarily using fiberoptic bronchoscopy. Muscle relaxants were avoided because airway management and tracheal intubation are anticipated to be difficult for patients with EB. Spontaneous respiration was maintained to minimize the risk of critical loss of airway control.

In our case, severe restriction of mouth opening precludes orotracheal intubation, making nasotracheal intubation the most viable option. However, we anticipated potential difficulty due to the risk of rapid oxygen desaturation and the need to avoid repeated intubation attempts. Therefore, a small-diameter tracheal tube (3 mm) was inserted nasally into the pharynx, and CPAP of 5 cm H₂O with an inspiratory pressure of 15 cm H₂O was applied to maintain oxygenation and allow continuous bronchoscopic visualization of the glottis.

The application of CPAP and positive pressure through the contralateral nostril may raise concern regarding pressure-induced mucosal injury or delayed laryngeal edema in patients with DEB. However, according to the International Consensus Best Practice Guidelines for Skin and Wound Care in Epidermolysis Bullosa, the fundamental pathological feature of EB is that the skin and mucosa blister or shear away in response to minimal everyday friction or mechanical trauma, rather than direct pressure [5]. Similarly, the Orphan Anesthesia recommendations for anesthesia management in EB emphasize that (1) prevention of blister formation relies primarily on minimizing friction and mechanical trauma, (2) the risk of blistering is particularly high at the pharyngeal and laryngeal levels during airway manipulation, and (3) newly formed blisters and wounds are mainly caused by friction and shearing forces, whereas the tissues are relatively tolerant of direct pressure [6].

The airway pressures applied in this case - approximately 5-10 cm H₂O during CPAP and up to 15 cm H₂O during inspiratory assistance - fall within the range of pressures that occur physiologically during vocal fold vibration in normal speech, with pressures of approximately 15 cm H₂O reported during strong phonation. Importantly, this patient was able to phonate and cough normally, similar to a healthy child, suggesting preserved tolerance of the laryngeal mucosa to physiological pressure changes.

Based on these considerations, we judged that the CPAP and inspiratory pressure settings used in this case were within a physiological range and were delivered via a noninvasive approach designed to minimize friction and shear forces. Consequently, the risk of pressure-induced mucosal injury or delayed laryngeal edema was considered limited in this carefully selected clinical context. The small-diameter tube with CPAP provided a secure airway, sufficient oxygenation, and adequate depth of sevoflurane anesthesia.

We fully acknowledge that bilateral nasal instrumentation may pose significant risks in patients with DEB, given the extreme fragility of the nasal, pharyngeal, and laryngeal mucosa. Potential complications include mucosal blistering, bleeding, sloughing, and acute or delayed airway obstruction. Simultaneous occupation of both nostrils can substantially limit rescue airway options in the event of bleeding, edema, or hypoxemia; therefore, this approach cannot be considered a standard airway management strategy in patients with DEB. However, careful consideration of potential risks is essential. This case suggests that the use of both nostrils for airway management, specifically nasopharyngeal CPAP delivered via a small-diameter tracheal tube, may serve as a useful adjunct in selected patients with recessive dystrophic epidermolysis bullosa (RDEB).

Previous reports have shown that in RDEB, oral mucosal involvement, including the lips, buccal mucosa, tongue, and floor of the mouth, occurs more frequently and with greater severity than nasal involvement [7]. Published data indicate oral involvement in approximately 53% of patients, whereas nasal involvement is reported in approximately 18% [8]. In the present case, severe mouth-opening restrictions and extensive oral mucosal involvement were present, leading us to judge that oral manipulation would pose a higher risk of mucosal injury than a carefully selected nasal approach. Accordingly, a nostril without visible lesions was selected, generous lubrication was applied, and an ultrathin bronchoscope was used to allow continuous visual confirmation of mucosal integrity throughout the procedure, thereby minimizing friction and shearing forces.

Preserving spontaneous breathing is generally considered to reduce the risks associated with airway management in patients with difficult airways. Nevertheless, when neuromuscular blocking agents are not used, vocal cord closure during tracheal intubation may occur and can itself result in increased shear stress and potential vocal cord injury. In our case, nasopharyngeal CPAP provided a secure airway, allowing the safe administration of a neuromuscular blocking agent and facilitating controlled, atraumatic fiberoptic nasotracheal intubation.

This report has several limitations. First, it describes a single case, and therefore, the safety and feasibility of this airway management strategy cannot be generalized to all patients with DEB. Second, the extent and severity of mucocutaneous involvement in EB vary widely among individuals, and careful case-by-case assessment remains essential. Finally, objective quantification of shear stress or airway pressure-related mucosal injury was not performed, and subtle or subclinical mucosal damage may not have been detected. Thus, further clinical experience and controlled studies are required before wider adoption can be recommended.

Overall, this case indicates that nasopharyngeal CPAP using a small-diameter tracheal tube could be a safe and effective adjunct in the airway management of patients with DEB, enabling the judicious use of muscle relaxants and successful tracheal intubation on the first attempt in a highly selected clinical setting.

## Conclusions

Airway management in patients with DEB is highly challenging because even minimal shear stress can cause blister formation in the airway. In this case, the use of nasopharyngeal CPAP with a small-diameter tracheal tube provided a secure airway and adequate oxygenation, which enabled safe nasotracheal intubation with the administration of muscle relaxants. This technique may be a valuable option for managing difficult pediatric airways in patients with EB.
